# A Clinical-Genetic Score to Identify Surgically Resected Colorectal Cancer Patients Benefiting From an Adjuvant Fluoropyrimidine-Based Therapy

**DOI:** 10.3389/fphar.2018.01101

**Published:** 2018-10-04

**Authors:** Elena De Mattia, Eva Dreussi, Marcella Montico, Sara Gagno, Chiara Zanusso, Luca Quartuccio, Salvatore De Vita, Michela Guardascione, Angela Buonadonna, Mario D’Andrea, Nicoletta Pella, Adolfo Favaretto, Enrico Mini, Stefania Nobili, Loredana Romanato, Erika Cecchin, Giuseppe Toffoli

**Affiliations:** ^1^Experimental and Clinical Pharmacology Unit, CRO Aviano National Cancer Institute, Istituto di Ricovero e Cura a Carattere Scientifico, Aviano, Italy; ^2^Scientific Directorate, Centro di Riferimento Oncologico di Aviano (CRO), IRCCS, Aviano, Italy; ^3^Department of Medical Area (DAME), Rheumatology Clinic, Santa Maria della Misericordia University Hospital, Udine, Italy; ^4^Medical Oncology Unit B, CRO Aviano National Cancer Institute, Istituto di Ricovero e Cura a Carattere Scientifico, Aviano, Italy; ^5^Medical Oncology Unit, “San Filippo Neri Hospital”, Rome, Italy; ^6^Medical Oncology Unit, University Hospital, Udine, Italy; ^7^Medical Oncology Unit, Ospedale di Treviso, Treviso, Italy; ^8^Department of Health Sciences, University of Florence, Florence, Italy

**Keywords:** colorectal cancer, fluoropyrimidines, interferon-γ, immune system, immunogenetics, adjuvant treatment

## Abstract

There are clinical challenges related to adjuvant treatment in colorectal cancer (CRC) and novel molecular markers are needed for better risk stratification of patients. Our aim was to integrate our previously reported clinical-genetic prognostic score with new immunogenetic markers of 5-year disease-free survival (DFS) to evaluate the recurrence risk stratification before fluoropyrimidine (FL)-based adjuvant therapy. The study population included a total of 270 stage II-III CRC patients treated with adjuvant FL with (FL + OXA, *n* = 119) or without oxaliplatin (FL, *n* = 151). Patients were genotyped for a panel of 192 tagging polymorphisms in 34 immune-related genes. The *IFNG*-rs1861494 polymorphism was associated with worse DFS in the FL + OXA (HR = 2.14, 95%CI 1.13–4.08; *P* = 0.020, *q*-value = 0.249) and FL (HR = 1.97, 95%CI 1.00–3.86; *P* = 0.049) cohorts, according to a dominant model. The integration of *IFNG*-rs1861494 in our previous clinical genetic multiparametric score of DFS improved the patients’ risk stratification (Log-rank *P* = 0.0026 in the pooled population). These findings could improve the discrimination of patients who would benefit from adjuvant treatment. In addition, the results may help better elucidate the interplay between the immune system and chemotherapeutics and help determine the efficacy of anti-tumor strategies.

## Introduction

For more than two decades 5-FU-based adjuvant chemotherapy has been the standard of care for patients with stage III and selected stage II CRC. Adding oxaliplatin (OXA) to therapy based on FLs (5-FU and capecitabine) further improves the disease-free (DFS) and OS rates in patients with stage III disease. However, the results from the QUASAR and MOSAIC trials demonstrated no further benefit when adding OXA to 5-FU in stage II patients, even those at high risk. This result supported that FLs monotherapy is the preferred treatment for a patient with stage II disease, even if the routine administration of adjuvant therapy is not recommended in these patients. Furthermore recent findings suggest that a shorter adjuvant chemotherapy (3 months instead of 6) could be considered for low-risk stage III disease (T1-3 N1 tumors) (Shi et al., 2017; Grothey et al., 2018). The introduction of the new targeted agents in the adjuvant setting has not brought any significant benefit (Dienstmann et al., 2015; Gustavsson et al., 2015; Loree and Cheung, 2016).

**GRAPHICAL ABSTRACT:**
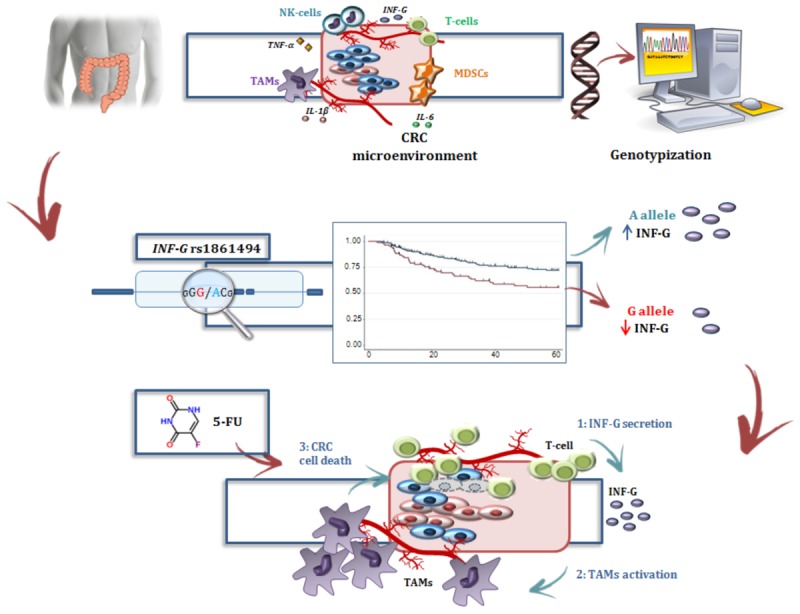


In this context, there is a need for new predictive markers, beyond the tumor stage, to select what patients will benefit from an adjuvant treatment and to better tailor treatment schemes and schedules. Mismatch repair (MMR) status has been proposed as a useful marker in patients with sporadic stage II CRC, together with additional parameters of high risk disease (age, T4 disease, tumor perforation, bowel obstruction, poor differentiation, perineural and/or lymphovascular invasion, and suboptimal number of lymph nodes examined) (Sargent et al., 2010). Pharmacogenetic studies have been also performed in order to evaluate the role of host genetic variants in the prediction of recurrence risk and response to adjuvant treatment with FLs and OXA. These investigations focused mainly on polymorphisms in genes encoding phase I and II enzymes (GSTP1), proteins involved in DNA repair (XRCC1 and XPD), folate-pathways (TYMS) and 5,10-methylenetetrahydrofolate reductase (MTHFR), and cell cycle control (CCDN1) (Libra et al., 2004; De Mattia et al., 2015; Smolle et al., 2015; Horvat et al., 2016; Kap et al., 2016). This group previously reported a clinical-genetic score based on the *MTHFR* polymorphism rs1801133, which significantly stratified a group of stages II–III CRC patients, receiving adjuvant FL-based treatment, according to DFS (Cecchin et al., 2015). However, the current methods for selecting CRC patients who would benefit from an adjuvant treatment are still sub-optimal.

The molecular and immune classification of CRC provided a new scenario for precision medicine, highlighting innovative prognostic and predictive factors for chemo and immunotherapies. Recently, the so-called immunoscore and tumor immune infiltration emerged as the best classifiers of CRC patients according to the prognosis and risk of tumor recurrence (Mlecnik et al., 2016; Pagès et al., 2018). The balance between pro- and anti-tumorigenic cytokines was found to modulate the inflammatory milieu in tumor tissues and to potentially contribute to CRC development, progression, and patient survival (Mager et al., 2016). An active interplay has been demonstrated to go on between these same cytokines, as interleukins (i.e., IL-1b, IL-6, IL-17, IL-15), TNFα, and interferon gamma (IFN-γ), and conventional chemotherapeutics, including 5-FU and OXA, eventually affecting the overall therapeutic outcome in patients undergoing anti-tumor treatments (Tesniere et al., 2010; Vincent et al., 2010; Apetoh et al., 2011; Cressman et al., 2012; De Mattia et al., 2013, 2018; Ni et al., 2013; Ghiringhelli and Apetoh, 2014; Guo et al., 2014; Wang et al., 2016; Wu et al., 2016; Hu et al., 2018).

This group previously reported how the germline profile of the leukocyte antigen gene family (*HLA*) can contribute to inter-individual differences in the therapy outcome of CRC patients receiving FL-containing therapy (De Re et al., 2014; Garziera et al., 2015). The present study was planned to broaden our immunogenetic analysis to genes encoding proteins involved in the immune system and related networks to highlight germ-line markers of DFS in two cohorts of stage II-III CRC patients receiving FL-based adjuvant therapy. The aim was to integrate these immunogenetic markers in our previously published clinical-genetic score (Cecchin et al., 2015) to improve the pre-treatment identification of patients who may benefit from an adjuvant FL-based treatment.

## Patients and Methods

### Patients’ Cohorts and Treatment

This retrospective study included a total of 270 patients, in two cohorts, with stages II–III CRC who were resected with a curative intent. All patients were treated with adjuvant FLs with or without OXA (Cecchin et al., 2013, 2015). All the patients were aged ≥18 years and had histologically confirmed stages II–III CRC, radiologically confirmed absence of distant metastases, a performance status (WHO) of 0–2, and normal bone marrow, renal and liver function. The FL + OXA cohort consisted of 151 CRC patients who underwent radical surgery between January 2004 and March 2011, and were treated with FOLFOX4 or CAPOX regimens, as previously reported (Haller et al., 2011; Cecchin et al., 2013). The FL cohort included 119 independent CRC patients who underwent radical surgery between May 1995 and May 2011, and subsequently received adjuvant FL-alone. Patients were treated with 5-FU/folinic acid according to the International Multicentre Pooled Analysis of Colon Cancer Trials (IMPACT) Investigators (1995), or capecitabine according to Twelves et al. (2005).

All the patients in the study were self-reported Caucasian. The study protocol complied with the ethical guidelines of the 1975 Declaration of Helsinki. The protocol was approved by the Comitato Etico Indipendente-Centro di Riferimento Oncologico di Aviano. All patients provided written informed consent for the genetic analysis before entering the study. All experiments were carried out in accordance with the relevant guidelines and regulations of Centro di Riferimento Oncologico di Aviano. Information on disease status and survival was obtained through the standard follow-up protocol for stages II/III surgically resected CRC patients. This consisted of a physical examination with routine blood tests, pulmonary X-ray, and abdominal ultra-sonography or computed tomography. Patients were assessed every 3 months during the treatment, every 6 months within the first 3 years, and then yearly (Cecchin et al., 2015).

### Candidate Genes and Polymorphism Selection

Target genes were selected on the basis of a literature search (PubMed-MEDLINE) focusing on genes encoding for proteins crucial for the regulation of the immune network and its potential interaction with chemotherapeutics to modulate antitumor response. For each candidate gene, genetic variants were chosen using the TagSNP approach. Genotype frequency data were downloaded from the HapMap website^1^ using the genomic coordinate defined according to the UCSC genome browser; the regions of interest were extended 5000 nucleotides further up- and downstream of the target gene to reasonably include all the regulatory regions. The filter parameters were the HapMap CEU database (release #27) and Minor allele frequencies (MAF) ≥ 0.05. The genotype data were then uploaded to the Tagger program implemented in Haploview^2^ (Broad Institute, Cambridge, MA, United States) to define the block of linkage polymorphisms at a stringency of *r*^2^ = 0.80. For each block, a TagSNP was picked, while prioritizing the polymorphisms with a predicted biological effect according to HaploRegv2 software^3^ and/or literature evidences. The highest priority was given to missense variants and polymorphisms previously associated with cancer or immune system activity. Next, variants located in a promoter or enhancer sequences or in regions bound by a transcription factor or other regulatory proteins were selected. At the end of this bioinformatics workflow, a set of 192 molecular markers in 34 candidate genes, correlated with immune system and cancer, were selected (**Supplementary Table S1**) and were introduced into the immunogenetic analysis.

### Genetic Analysis

Genomic DNA was extracted from peripheral blood using the High Pure PCR Template Preparation Kit (Roche Diagnostics GmbH, Mannheim, Germany). DNA samples were genotyped using the Illumina BeadXpress platform based on Golden Gate chemistry. A 192-plex Illumina VeraCode GoldenGate Genotyping Assay (Illumina, Inc., San Diego, CA, United States) was developed using the Assay Design Tool (ADT) available on the Illumina website^4^. The bioinformatics tool assigned a final score (ranging from 0 to 1.1) and designability score (ranging from 0 to 1) for each variant and these scores correlated with the quality and robustness of the assay. Only assays with a high final score (≥0.7) and optimal designability (=1) were considered compatible with successful GoldenGate genotyping and were introduced into the final custom panel. Samples were prepared for the analysis according to the manufacturer’s protocol. VeraScan software (version 2.0) was employed for fluorescence detection and the GenomeStudio V2011.1 tool (Illumina, Inc.) was used for genotype clustering with a polymorphism call-threshold of 0.25 (on a scale of 0–1). The clusters generated by the program were manually reviewed to ensure high quality data. The control dashboard was checked to evaluate the overall quality of the analyses and to exclude samples with low performance. Sample replicates were introduced into each analysis to assess the robustness of the output records and to provide duplicate data to aid in the redefinition of clustering. Only the polymorphisms with a call rate > 80% were retained in the final report. More details about the analytical procedures are available upon request.

### Study Design and Statistical Analysis

The main study endpoint was DFS. A stepwise selection of significant markers of DFS was performed. The first step consisted of screening the entire set of polymorphisms for associations with DFS in the FL + OXA cohort (151 subjects), that was selected as discovery cohort due to the larger sample size providing stronger statistical power. Only the polymorphisms significantly associated with DFS in the first cohort (*P* < 0.05) were genotyped for association with DFS in the FL cohort (119 subjects), applying the same genetic model. The genetic variants with significant (*P* < 0.05) associations with DFS in both cohorts were integrated in the previously published multi-parametric score of DFS in the pooled population. The score included four previously identified prognostic markers (*i.e.*, *MTHFR*-rs1801131 polymorphism, gender, primary tumor site, and stage). As a secondary analysis, the genetic variants with a concordant effect on DFS in the FL + OXA and FL cohorts were further evaluated for their association with OS in the pooled population.

The effect of the polymorphisms on DFS or OS was assessed through HRs and corresponding 95% CIs, estimated by COX proportional hazard models. The HRs were adjusted for gender, age, primary tumor site, and tumor TNM stage. Dominant, recessive, and additive genetic models were considered; the best-fitting model was selected according to the Wald χ^2^ test. A *P-*value < 0.05 (two-sided) was adopted as the significance threshold. To assess the effect of the multiple testing in the FL + OXA cohort, where the genetic markers have been selected, a *q*-value (FDR-adjusted *P*-value) was evaluated (Benjamini and Hochberg, 1995). Survival analysis was performed by the Kaplan–Meier method, and the log-rank test was used to test the differences between groups. The DFS was calculated from the time of surgery to the most recent, available medical examination or the date of recurrence. The OS was measured from the date of surgery to the most recent follow-up or the date of death. Patient follow-up was truncated at 5 years.

## Results

### Patients Characteristics and Genotyping

The main demographic and clinical characteristics of the two cohorts are reported in **Table 1**. The FL + OXA and FL cohorts were well-balanced for gender, age, primary tumor site, and the FL administration (5-FU or capecitabine); while the tumor stage distribution at diagnosis was different between the two cohorts with a higher prevalence of stage III CRC in the FL + OXA group.

**Table 1:** Demographic and clinical characteristics of the two patients cohorts included in the study (discovery and replication cohort).

	FL + OXA (*n* = 151)	FL (*n* = 119)
**Characteristic**	***N* (%)**	***N* (%)**
**Sex**		
Male	79 (52.3)	68 (57.1)
Female	72 (47.7)	51 (42.9)
**Age (median, IQR)**	62 (53–68)	67 (58–74)
**Primary tumor site**		
Colon	118 (78.1)	89 (74.8)
*Right*	41 (27.2)	30 (25.2)
*Left*	72 (47.7)	51 (42.9)
*Transverse*	5 (3.3)	8 (6.7)
Rectum	33 (21.9)	30 (25.2)
**Stage at diagnosis**^*a*^		
II	20 (13.2)	55 (46.2)
III	131 (86.7)	64 (53.8)
**Fluoropyrimidine**		
5-fluorouracil	119 (78.8)	97 (81.5)
capecitabine	32 (21.2)	22 (18.5)
**DFS at 5 years**		
Number of recurrences^*b*^	39 (25.8)	35 (29.4)
DFS rate (95% CI)	65.9% (55.9–74.1)	67.8% (58.1–75.8)
**OS at 5 years**		
Number of deaths^b^	20 (13.2)	20 (16.8)
Survival rate (95% CI)	82.2% (73.5–88.3)	80.5% (71.3–87.0)

*^a^Tumor node metastasis scale (TNM). ^b^The number of events that occurred during the 5 years of follow-up. DFS, disease-free survival; IQR, interquartile range; OS, overall survival; 95% CI: 95% confidence interval.*

Genotyping was successful for 164/192 assays by a custom-designed GoldenGate Genotyping analysis (BeadXpress, Illumina). Twenty-eight markers failed at the analysis and were excluded from the study. The average genotype call rate was 0.98 (range: 0.84–1.00). All 270 patients eligible for the study were successfully genotyped with an average call rate of 0.98 (range: 0.68–1.00). The average concordance rate was 100% for replicated samples included in the analyses.

### Markers of Disease-Free Survival

In the FL + OXA cohort, nine polymorphisms in *SMAD3*, *FOXO3*, interferon gamma (*IFNG*), transforming growth factor beta receptor 1 and -2 (*TGFBR1*/*-2*), signal transducer and activator of transcription 5A and 5B (*STAT5A*/*–B*), and one angiogenesis regulator (*VEGFA*) were associated with the patients’ DFS (**Table 2**). Of the nine identified markers, six were associated with an increased risk of recurrence, with HRs ranging from 2.40 to 4.57, and the remaining three were associated with a lower risk of recurrence, with HRs ranging from 0.33 to 0.53. FDR analysis pointed out that the nine markers associated with DFS (*P* < 0.05) in the FL + OXA cohort had a *q*-value below 0.250, ranging from 0.032 to 0.249.

**Table 2:** Hazard ratio (HR) and 95% confidence interval (95% CI) for 5-years disease-free survival (DFS) in the FL + OXA (*n* = 151), FL (*n* = 119), and pooled (*n* = 270) cohorts of stages II–III colorectal patients according to gene polymorphisms (SNPs).

Gene	SNP	Base change		FL + OXA (*n* = 151)			FL (n = 119)	
			**Mod**	**HR (95% CI)^*a*^**	***p*-value**	***q*-value^*b*^**	**HR (95% CI)^a^**	***p*-value**
*SMAD3*	rs11636161	G > A	Rec	2.81 (1.38–5.71)	0.004	0.108	1.67 (0.81–3.45)	0.168
*SMAD3*	rs1545161	A > G	Add	0.53 (0.33–0.85)	0.008	0.185	1.03 (0.65–1.64)	0.892
*FOXO3*	rs12203787	G > C	Dom	0.33 (0.13–0.86)	0.024	0.203	1.37 (0.52–3.64)	0.522
***IFNG***	**rs1861494**	**A > G**	**Dom**	**2.14 (1.13–4.08)**	**0.020**	**0.249**	**1.97 (1.00–3.86)**	**0.049**
*VEGFA*	rs2146323	C > A	Dom	0.43 (0.22–0.84)	0.014	0.203	0.86 (0.41–1.80)	0.688
*TGFBR1*	rs928180	A > G	Dom	2.40 (1.21–4.75)	0.012	0.194	1.63 (0.61–4.40)	0.333
*TGFBR2*	rs1346907	A > G	Rec	2.49 (1.24–4.98)	0.010	0.194	0.57 (0.18–1.77)	0.329
*STAT5A*	rs7217728	A > G	Rec	3.58 (1.71–7.48)	0.001	0.032	0.58 (0.17–2.06)	0.404
*STAT5B*	rs8080122	G > A	Rec	4.57 (1.9–10.99)	0.001	0.032	0.58 (0.17–2.06)	0.402

*95% CI, 95% confidence interval; FL, fluoropyrimidines; HR, hazard ratio; OXA, oxaliplatin; SNP, single nucleotide polymorphism. ^a^Estimated from Cox model, adjusted for gender, age, cancer site, stage at diagnosis. ^b^False discovery rate-adjusted p-value. Only the associations with P < 0.05 are reported in the FL + OXA set and replicated in the FL cohort. Markers with the same predictive effect and below the cut-off for statistical significance (P < 0.05) in all cohorts are evidenced in bold.*

**FIGURE 1:**
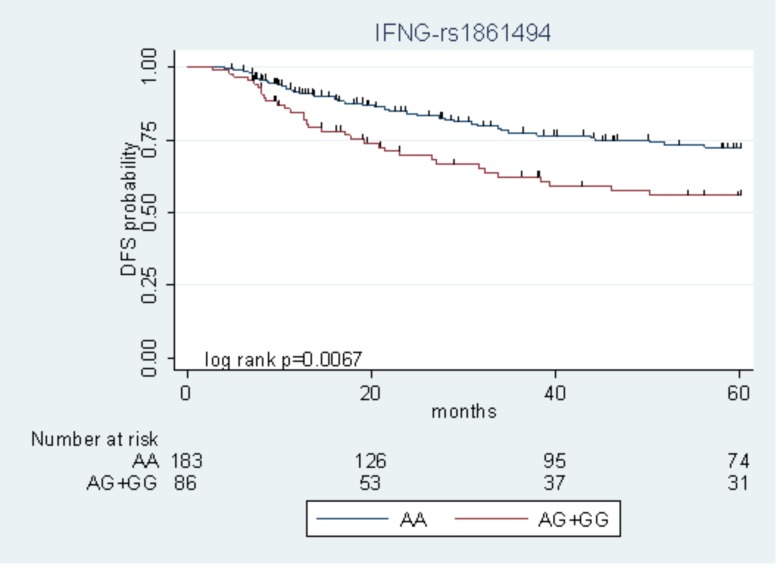
Kaplan–Meier estimates of disease-free survival (DFS) according to the *IFNG*-rs1861494 polymorphism in the pooled group of patients.

Among the nine polymorphisms highlighted in the FL + OXA cohort, *IFNG*-rs1861494 was successfully replicated in the FL cohort. Particularly, the G allele of *IFNG*-rs1861494 was significantly associated with a worse DFS in the FL + OXA (HR = 2.14, *P* = 0.020, *q*-value = 0.249) and FL (HR = 1.97, *P* = 0.049) cohorts, according to a dominant model. When considering the pooled population of patients (FL + OXA plus FL), the association was more significant (HR = 1.91, *P* = 0.006). The DFS Kaplan–Meier curves, according to the *IFNG*-rs1861494 genotype, in the pooled population are shown in **Figure 1**. At 5-years follow-up, 72.2% of patients harboring the rs1861494-AA genotype were free of tumor recurrence (95% CI: 64.0–78.8) versus 55.7% (95% CI: 43.5–66.4) of those carrying the rs1861494-AG/GG genotype (Log-rank *P* = 0.0067).

Three markers out of nine, *SMAD3*-rs11636161, *TGFBR1*-rs928180, and *VEGFA*-rs2146323, had an effect on DFS that was in the same direction in both the FL + OXA and FL cohorts according to the same genetic model; although, it was not significant (*P* > 0.05) in the FL cohort. Five markers out of nine (*i.e.*, *SMAD3*-rs1545161, *FOXO3*-rs12203787, *TGFBR2*-rs1346907, *STAT5A*-rs7217728, *STAT5B*-rs8080122) selected for their significant impact (*P* < 0.05) on DFS in the FL + OXA cohort, displayed an opposite effect, although not significant (*P* > 0.05), in the FL cohort. The genotype distribution of the nine markers highlighted for their significant effect on DFS in the discovery cohort is reported in **Supplementary Table S2**. MAFs were checked and found to be in line with the data reported for the Caucasian population^5^.

### Markers of Overall Survival

**FIGURE 2:**
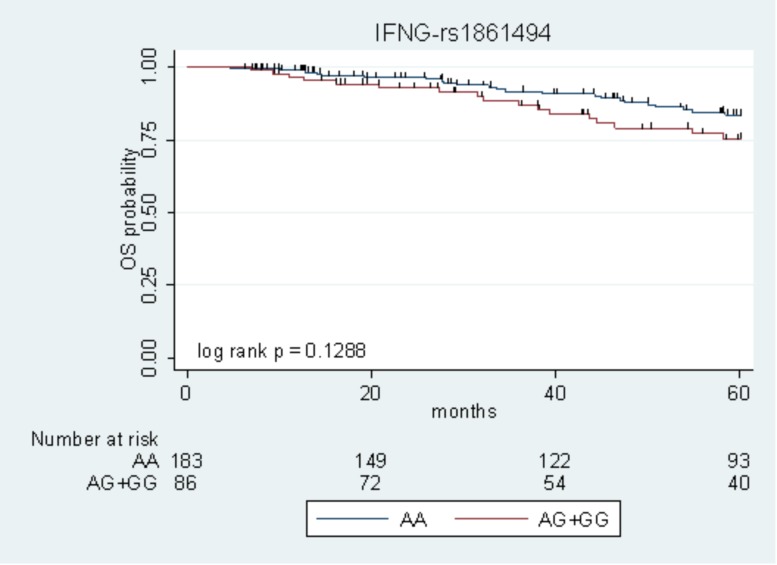
Kaplan–Meier estimates of overall survival (OS) according to the *IFNG*-rs1861494 polymorphism in the pooled population.

The only marker associated with DFS with *P* < 0.05 in the two cohorts (*IFNG*-rs1861494) was tested for its effect on OS, according to the same genetic model. Due to the low number of events, the survival analysis was performed on the pooled population of patients. The *IFNG-*rs1861494-G allele, associated with low DFS, exhibited a tendency toward an increased risk of death (HR = 1.69, 95% CI: 0.90–3.19, *P* = 0.105). Kaplan–Meier curves of OS according to the *IFNG*-rs1861494 variant are shown in **Figure 2**.

At 5-years follow-up, the percentage of patients who were still alive was 83.5% (95% CI: 76.1–88.8) among those harboring the rs1861494-AA genotype vs. 75.3% (95% CI: 63.1–84.0) among those carrying the rs1861494-AG/GG genotype (Log-rank *P* = 0.1288).

### Risk Model in the Pooled Population

**FIGURE 3:**
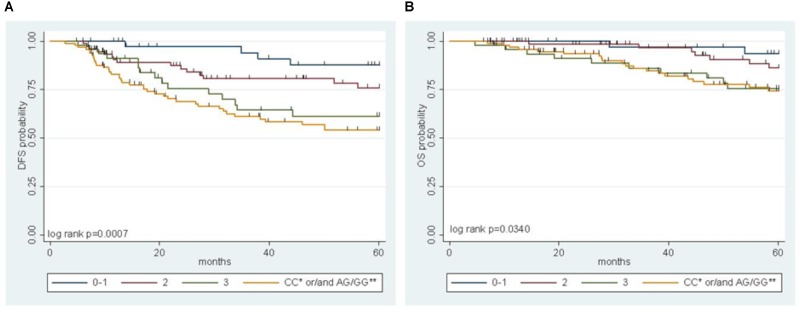
Multiparametric score of disease free survival (DFS) **(A)** and overall survival (OS) **(B)** in the pooled group of patients according to an increasing number of clinical (gender, tumor site, and stage) and genetic (*MTHFR*-rs1801131, *IFNG*-rs1861494) risk factors. ^∗^refers to *MTHFR*-rs1801131 variant; ^∗∗^ refers to *IFNG*-rs1861494 variant.

A multiparametric score of DFS integrating the genetic *MTHFR*-rs1801131 marker with clinical factors (*i.e.*, gender, primary tumor site, and tumor stage) was previously developed, where the *MTHFR*-rs1801131-CC genotype (vs. rs1801131-AA/AC genotype), male sex (vs. female), colon primary tumor site (vs. rectum), and tumor stage III (vs. stage II) were considered negative prognostic factors for DFS (Cecchin et al., 2015). In the present study, the *IFNG*-rs1861494 polymorphism, selected by a stepwise procedure as a significant marker of DFS, was integrated in the risk model to improve its prediction power. The detrimental prognostic effect of carrying 0 to 3 non-genetic features, according to the previous study, was compared with the effect of carrying both the detrimental genetic factors (*i.e.*, *MTHFR*-rs1801131-CC or *IFNG*-rs1861494-AG/GG) in the pooled population (**Figure 3A**, Log-rank *P* = 0.0007).

A significant increase in the risk of recurrence according to the number (0–1 vs. 2 vs. 3) of clinical-demographic risk parameters in patients with a favorable genetic background (*i.e.*, *MTHFR*-rs1801131-AA/AC or *IFNG*-rs1861494-AA genotype) was observed, in line with previous data (Cecchin et al., 2015). Nevertheless, carrying either one of the detrimental genetic factors (*i.e.*, *MTHFR*-rs1801131-CC or *IFNG*-rs1861494-AG/ GG genotype) discriminated the patients with the worse prognosis, independently from other non-genetic characteristics (**Table 3**).

**Table 3:** Hazard ratio (HR) and 95% confidence interval (95% CI) for 5-yeras disease free survival (DFS) and overall survival (OS) in the pooled group of patients according to an increasing number of clinical (gender, tumor site and stage) and genetic (*MTHFR*-rs1801131, *IFNG*-rs1861494) risk factors.

Number of genetic risk factorsˆ	Number of clinical risk factors	Number of patients§	HR (95% CI)	
			**DFS**	**OS**
0	0–1	40	1	1
0	2	78	2.28 (0.78–6.67)	2.08 (0.44–9.85)
0	3	50	3.91 (1.34–11.39)	4.65 (1.03–21.03)
≥1	Any	99	5.20 (1.92–14.08)	4.81 (1.14–20.38)

*ˆ0 = rs1801131-AA/AC and rs1861494-AA; 1 = rs1801131-AA/AC and rs1861494-AG/GG or rs1801131-CC and rs1861494-AA; 2 = rs1801131-CC and rs1861494-AG/GG. ^§^ Three patients were not genotyped for both the MTHFR-rs1801131 and IFNG-rs1861494 variants. DFS, disease-free survival; 95% CI, 95% confidence interval; HR, hazard ratio; OS, overall survival.*

The same result was obtained also when only pathological stage III was considered (Log-rank *P* = 0.0026, **Supplementary Figure S1**). The performance of the multiparametric score in stratifying patients with different OS outcomes was then evaluated. This analysis demonstrated the same trend observed for DFS in the different classes of patients (**Figure 3B**, Log-rank *P* = 0.0340; **Table 3**). The distribution of the clinical-demographic risk factors in the two cohorts of patients harboring at least one detrimental genetic factor (*MTHFR*-rs1801131-CC or *IFNG*-rs1861494-AG/GG genotype) or a favorable (*MTHFR*-rs1801131-AA/AC and *IFNG* rs1861494-AA genotype) genetic background was well-balanced (χ^2^ for association *P* = 0.951).

## Discussion

To date, pathologic tumor staging remains the key determinant for choosing adjuvant treatment in CRC even if a considerable stage-independent outcome variability is observed. Therefore, there is still a need for prognostic/predictive markers to better stratify patients in the adjuvant setting. The main finding of this study was the identification of *IFNG*-rs1861494 as a marker of DFS in two independent cohorts of patients, treated with FL with or without OXA. In the pooled set of patients, the same marker showed also a trend toward shorter OS. The *IFNG*-rs1861494 polymorphism was successfully integrated in a previously published clinical-genetic score including other clinical risk factors (*i.e.*, gender, primary tumor site, and tumor stage) and the patient’s genotype for *MTHFR*-rs1801131. It came out that carriers of the *MTHFR*-rs1801131-CC or *IFNG*-rs1861494-AG/GG genotype had the worst prognosis than all the rest of the patients and this was independent from the other risk factors, including tumor stage.

*IFNG* encodes for interferon-γ (IFN-γ), also known as type II interferon, a pro-inflammatory cytokine that participates in the regulation of both innate and adaptive immunity against pathogens or cancer cells (Schroder et al., 2004; Kosmidis et al., 2018). This cytokine induces a protective and anti-tumor response in CRC patients (Evans et al., 2006; Kantola et al., 2012; Ganapathi et al., 2014); accordingly, reduced expression of IFN-γ in peripheral blood mononuclear cells of CRC patients could contribute to CRC progression and recurrence (Ganapathi et al., 2014). In addition to the importance of an adequate IFN-γ signal for maintaining a tumor-prohibitive environment, a significant interaction between this cytokine and the mechanism of action of 5-FU has been reported. *In vitro* and *in vivo* data from experimental tumor models have demonstrated that 5-FU has the capacity to eliminate the MDSCs that contribute to the immune tolerance of cancer by inhibiting the function of CD8(+) T cells. This mechanism was reported to enhance the secretion of IFN-γ by tumor specific CD8(+) T cells and to promote T-cell dependent antitumor responses (Vincent et al., 2010; Apetoh et al., 2011). Other *in vitro* and *in vivo* data (Patras et al., 2016; Malesci et al., 2017) further indicated there was an interplay between 5-FU and the TAMs, another class of immune cells whose activity is partially regulated by IFN-γ (Poh and Ernst, 2018), in determining CRC cell death and the efficacy of adjuvant 5-FU-based therapy. Furthermore, a direct interaction between 5-FU and IFN-γ was observed by *in vitro* analyses that showed sensitization of human colon carcinoma cell lines to 5-FU that was induced by the cytokine through modulation of the expression of specific genes involved in apoptosis regulation (Adachi et al., 1999; Schwartzberg et al., 2002).

**FIGURE 4:**
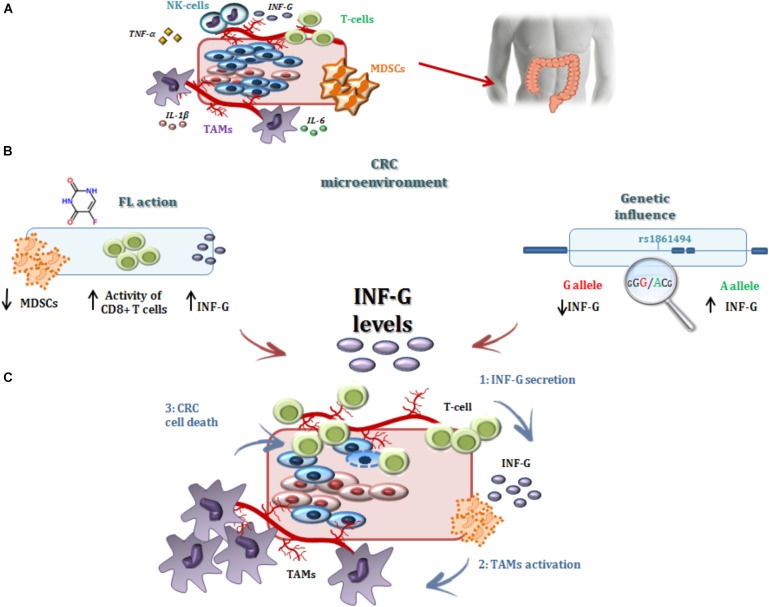
Interferon-γ in the modulation of the anti-tumor efficacy of fluoropyrimidine-based treatment. **(A)** Different immune cells, such as T-cells, MDSCs, TAMs, and a complex network of cytokines that mediate cell interactions, significantly contribute to the formation of the CRC microenvironment. **(B)** Treatment with FLs, as well as the genetic profile, influenced the level of IFN-γ, a key cytokine for shaping the tumor microenvironment and determining the antitumor response to treatment. Particularly, FLs induce MDSC apoptosis, promoting the activity of CD8+ T cells and a higher secretion of IFN-γ. On the other hand, the *IFNG*-rs1861494 polymorphism modulates IFN-γ expression: the G-allele introduces a new CpG methylation dinucleotide site that leads to inferior production of this cytokine; whereas the A-allele is correlated with its enhanced expression. **(C)** It could be speculated that higher levels of IFN-γ (1), as the result of increased release by immune cells activated through a FL-dependent mechanism in a compliant genetic context (*i.e.*, *IFNG*-rs1861494-AA genotype), leads to an increased activation of TAMs (2) that finally promote CRC cell death (3) and potentially a more efficient FL-based treatment. The presence of the rs1861494-AG/GG genotype could contribute to this hypothesized mechanism with a negative impact of the FL-based treatment outcome. CRC, colorectal cancer; FLs, fluoropyrimidines; IFN-G, interferon gamma; IL-1β/-6, Interleukin-1β/-6; MDSCs, myeloid derived suppressor cells; NK, natural killer; TAMs; tumor associated macrophages, T-cell, T-lymphocyte; TNFα, tumor necrosis factor alpha.

In the present study, the *IFNG*-rs1861494-G allele was associated with an increased risk of CRC recurrence after FL-based adjuvant therapy. The phenotypic consequences of this polymorphism, located within a conserved regulatory region of the third intron of *IFNG*, is well-characterized. Specifically, functional analyses showed that the rs1861494 T to C change (corresponding to A to G in the current analysis) introduces a new CpG methylation dinucleotide site that changes the methylation pattern of the gene, resulting in a distorted transcription factor binding to this region and an altered IFN-γ transcriptional level. Consequently, the common rs1861494-A allele was correlated with enhanced expression and secretion of IFN-γ; while, the minor rs1861494-G allele correlated with inferior production of the cytokine (Gonsky et al., 2014). These functional data are in line with the results of the present work. A decreased IFN-γ level, associated with the rs1861494-G allele, could both deregulate the anti-proliferative activity of IFN-γ and alter the 5-FU cytotoxicity toward cancer cells (**Figure 4**), resulting in an increased risk of CRC recurrence and poor prognosis, as reported by the current paper.

In the present study, the *IFNG*-rs1861494 genotype was also combined with other genetic (*i.e.*, *MTHFR*-rs1801131 genotype) and non-genetic factors (gender, primary tumor site, stage) to integrate a previously developed risk model for DFS (Cecchin et al., 2015). The incorporation of *IFNG*-rs1861494 in the multiparametric score strongly improved the stratification of patients according to their different recurrence risks or survival profiles. MTHFR is a key enzyme for intracellular folate homeostasis and metabolism, catalyzing the irreversible conversion of 5,10-methylenetetrahydrofolate, required for DNA synthesis, to 5-methyltetrahydrofolate, the primary methyl donor indispensable for nucleic acid methylation (Toffoli et al., 2003; De Mattia and Toffoli, 2009; De Re et al., 2010). The missense *MTHFR*-rs1801131 polymorphism (1298A > C; Glu29Ala) was associated with decreased enzyme activity and higher 5-FU cytotoxicity. The influence of the *MTHFR* genotype on FL sensitivity could be related to mechanisms as a change in the distribution of folate pools, a modification in DNA methylation patterns as well as an influence on the development of microsatellite instable (MSI) CRC (Cecchin et al., 2015). A genetic MTHFR deficiency, and the related disruption in folate metabolism, may impact the immune response by altering the expression of inflammatory mediators, including IFN-γ (Mikael et al., 2013; Meadows et al., 2014). In this respect, the risk score combining the two functionally relevant variants in *MTHFR* and *IFNG* optimally integrated the impact of the markers on the same biological pathway. This finding further corroborates the effectiveness of combining genetic and non-genetic factors and of simultaneously evaluating the joint effect of multiple genetic markers when looking for new prognostic biomarkers in cancer (Di Francia et al., 2010; De Mattia et al., 2015).

The integration of *IFNG*-rs1861494 in the clinical-genetic score allowed the identification of a larger group of patients with a bad prognosis, further refining the stratification of patients and suggesting different therapeutic approaches tailored to the patient’s genetic profile. Moreover, when looking at patients with the same tumor stage, this score was still able to significantly stratify patients into different prognosis groups (**Supplementary Figure S1**). This demonstrates not only that the score is tumor stage independent, but first of all that a classification of CRC patients based only on tumor stage is no longer appropriate. A large inter-individual variability in the response to adjuvant treatment was reported among patients of the same stage (Sargent et al., 2010; Shi et al., 2017) and additional diagnostic parameters are needed to personalize patients’ therapeutic strategies. The present study highlights that, within the same tumor stage, there are classes of patients with extremely different prognoses and that patients’ germline variations play an important role. These findings should be considered when planning adjuvant treatment.

In the preset study, some polymorphisms, *SMAD3*-rs11636161, *TGFBR1*-rs928180, and *VEGFA*-rs2146323, *SMAD3*-rs1545161, *FOXO3*-rs12203787, *TGFBR2*-rs1346907, *STAT5A*-rs7217728, *STAT5B-*rs8080122, had a significant effect on DFS in the discovery cohort, that was not replicated in the replication cohort. This lack of replication does not exclude a regimen-specific (FL-alone or FL + OXA) prognostic effect. However, no adequate clinical and molecular information are available to provide insights of this possible interaction. It could also be that the lower number of patients in the replication cohort prevents to observe an effect, that could have been significant with a larger replication cohort. It must therefore pointed out that the predictive value on FL-based therapy outcome of these genetic variants should be considered as exploratory but is worthy of further evaluations.

Some limitations of the present study need to be considered. First, a number of clinical and molecular features that are well-known to influence the prognosis of CRC patients treated with post-operative chemotherapy were not evaluated in the current analysis. It should be noted that, when controlling the analysis for multiple testing, the FDR for the association between *IFNG*-rs1861494 and DFS was 25.0%, pointing out that the study results should be considered only as hypothesis-generating. Despite this, replicating a significant association in an independent set of patients (i.e., the FL cohort), as in the present study, strengthens the reliability of the data and the interest in further clarifying its potential clinical implication.

The results from our study help better define the complex and multifaceted mechanism of action of FL and its crucial interplay with IFN-γ. The findings of the current study further confirm the pivotal role of the immune system in determining the effectiveness of the FLs as well as the interaction between the immune system and the anticancer drug itself. This interaction between chemotherapy and immune pathway in cancer is of great interest due to the success of immunotherapy for different tumor types. These data could contribute to improving the clinical use of the novel immune checkpoint inhibitors (i.e., anti-programmed cell death protein 1, PD-1) (Toh et al., 2016; Passardi et al., 2017; Arora and Mahalingam, 2018) by suggesting a potential synergism between immunotherapy and the traditional chemotherapeutics (Van Der Kraak et al., 2016; Di Franco et al., 2017; Emambux et al., 2018). In this context, the discovery of novel markers that predict the impact of chemotherapy on tumor immunity could be important for selecting patients who could benefit from immune modulators in combination with anticancer agents.

## Conclusion

The risk model of the present study, where the genetic features had a high prognostic capacity even in a pathologic stage-independent manner, could represent a useful tool for the clinician to optimize adjuvant treatment in CRC patients. Indeed, the identification of novel markers that can better stratify patient’s risk is of great importance to avoid more extensive interventions and the associated toxicity, inconvenience, and cost for low-risk patients. On the other hand, these markers could help intensify therapy for those who are at higher risk for recurrence.

## Author Contributions

EDM and EC were involved in designing the study, critically revising the results, and preparing the manuscript. ED participated in the creation of the tables and figures. MM was involved in the statistical analysis and interpretation of data. CZ, ED, and LR were involved in the molecular analysis. SG, LQ, and SDV participated in the marker selection, genotyping assay development, and collection of genotyping data. MG, AB, MD, NP, AF, EM, and SN participated in the patient enrollment and in the collection of clinical data. GT was the guarantor. All authors reviewed the manuscript.

## Conflict of Interest Statement

The authors declare that the research was conducted in the absence of any commercial or financial relationships that could be construed as a potential conflict of interest.

## Footnotes

1 http://hapmap.ncbi.nlm.nih.gov/

2 http://www.broad.mit.edu/mpg/haploview

3 https://pubs.broadinstitute.org/mammals/haploreg/haploreg_v2.php

4 https://illumina.com

5 http://www.ncbi.nlm.nih.gov/snp

## Abbreviations

5-FU: 5-fluorouracil
95% CI: 95% confidence interval
CCDN1: Cyclin D1
CRC: colorectal cancers
DFS: disease-free survival
FDR: false discovery rate
FL: fluoropyrimidine
FOXO3: forkhead box O3
GSTP1: glutathione-*S*-transferase P1 isoform
HLA-G: leukocyte antigen
HR: hazard ratio
IFN-γ (*IFNG*): interferon-gamma
*IFNGR*1/-2: interferon gamma receptor 1/-2
IL-X: interleukin-X
MAF: minor allele frequency
MDSC: myeloid-derived suppressor cell
MTHFR: 5,10-methylenetetrahydrofolate reductase
OS: overall survival
OXA: oxaliplatin
SMAD3: SMAD family member 3
STAT1: signal transducer and activator of transcription 1
STAT5A/–B: signal transducer and activator of transcription 5A/-B
TagSNP: tagging polymorphism
TAM: tumor-associated macrophage
TGFBR1/-2: transforming growth factor beta receptor 1/-2
TIL: tumor infiltrating lymphocyte
TNFα: tumor necrosis factor alpha
TYMS: thymidylate synthase
VEGFA: vascular endothelial growth factor A
XPD: Xeroderma pigmentosum group D
XRCC1: X-ray repair complementing defective repair in Chinese hamster cells 1.
