# Supplementary material for: A Clinical-Genetic Score to Identify Surgically Resected Colorectal Cancer Patients Benefiting From an Adjuvant Fluoropyrimidine-Based Therapy
Source: Front Pharmacol. 2018 Oct 4;9:1101. doi: 10.3389/fphar.2018.01101 (PMC6180157; doi:10.3389/fphar.2018.01101)
Supplement: Supplementary file 1 [file Data_Sheet_1.PDF]

Title: A clinical-genetic score to identify surgically resected colorectal cancer patients benefiting from an adjuvant fluoropyrimidine-based therapy

Authors: Elena De Mattia[*1], Eva Dreussi[1], Marcella Montico[2], Sara Gagno[1], Chiara Zanusso[1], Luca Quartuccio[3], Salvatore De Vita[3], Michela Guardascione[1], Angela Buonadonna[4], Mario D'Andrea[5], Nicoletta Pella[6], Adolfo Favaretto[7], Enrico Mini[8], Stefania Nobili[8], Loredana Romanato[1], Erika Cecchin[#*1] and Giuseppe Toffoli[#1]

[#]Cecchin E. and Toffoli G. share last authorship

Correspondence to:

***Dr. Elena De Mattia PhD,** Clinical and Experimental Pharmacology, CRO- National Cancer Institute, Via Franco Gallini n. 2, 33081 Aviano (PN) –Italy. e-mail: edemattia@cro.it; Phone number: +39-0434-659765; Fax: +39-(0)434-659799

***Dr. Erika Cecchin PhD,** Clinical and Experimental Pharmacology, CRO- National Cancer Institute, Via Franco Gallini n. 2, 33081 Aviano (PN) –Italy. email: ececchin@cro.it; Phone number: +39-0434-659667; Fax: +39-(0)434-659799

Supplementary Table S1: Candidate genes and related Tagging polymorphisms selected for pharmacogenetic analysis.

Gene	Tagging polymorphisms
CD276 (B7-H3)	rs3825859
	rs8038465
	rs2127015
	rs10083681
CXCR7	rs10179774
	rs7559855
	rs34135799
	rs10184764
FAS	rs3740286
	rs7097467
	rs1800682
	rs2234978
	rs9658727
	rs4406737
	rs9658706
	rs982764
	rs983751
FOXO3	rs2153960
	rs12203787
	rs7762395
	rs2802288
	rs9384683
	rs9486902
	rs13207511
	rs12196996
	rs1536057
	rs3800230
	rs7746906
	rs2294019

	rs2232365
	rs3761548
	rs3761547
IFNG	rs1861494
IFNGR1	rs9376269
	rs10457655
IFNGR2	rs9808685
	rs9808753
	rs1532
	rs2834213
	rs2834211
IL15RA	rs17320853
	rs8177613
	rs8177633
	rs8177654
	rs2296141
	rs1998521
	rs3136626
	rs2228059
	rs7910212
	rs3736862
IL17A	rs1892280
	rs2275913
	rs10484879
IL17F	rs607175
	rs12210153
	rs641701
	rs2064331
	rs9463772
	rs763780
IL2RA	rs12722489
	rs1107345

	rs706778
	rs10905656
	rs2256774
	rs6602398
	rs12722588
	rs3118470
	rs11256448
	rs10905668
	rs4749920
IL2RB	rs2284033
	rs3218266
	rs84460
	rs3218322
	rs3218258
	rs228942
IL2RG	rs12857595
IL8	rs2227306
MIF	rs2000466
	rs738806
	rs875643
	rs1007888
MMP3	rs679620
	rs569444
	rs683878
PRDM1	rs4946722
	rs1984224
	rs573869
	rs811925
	rs6923608
SMAD3	rs2118613
	rs11636161
	rs17293632

	rs12917612
	rs4776338
	rs2033785
	rs17228212
	rs4776343
	rs12708492
	rs1545161
	rs4147358
	rs3743343
	rs12916733
	rs718663
	rs2033787
	rs2289263
	rs12914140
	rs16950635
	rs991157
	rs7162912
	rs7179840
	rs4776887
	rs9302242
SMAD3	rs11856909
SMAD4	rs12457540
	rs948588
	rs10502913
STAT3	rs3744483
	rs9891119
	rs8069645
	rs744166
	rs17405722
STAT5A	rs1053023
	rs7217728
STAT5B	rs8080122

STAT6	rs703817
	rs3024979
	rs3024974
	rs1059513
	rs167769
TGFBR1	rs10988716
	rs928180
TGFBR2	rs3773632
	rs1841528
	rs5020833
	rs2276767
	rs3773658
	rs9867701
	rs12487185
	rs9790268
	rs4955104
	rs4583693
	rs995435
	rs3773649
	rs6550004
	rs4955212
	rs764522
	rs1346907
	rs876688
	rs4522809
	rs1078985
	rs3773662
	rs17025857
	rs11709624
	rs11924422
	rs1835538
	rs9310940

	rs1991657
TIMP1	rs6609533
	rs6609534
TIRAP	rs8177376
	rs10893493
	rs625413
	rs1893352
	rs1786704
TLR10	rs11466617
	rs7660429
	rs11096955
	rs11096957
	rs11725309
	rs11466657
TLR3	rs11721827
	rs5743303
	rs7657186
	rs3775291
TLR4	rs1927911
	rs11536898
	rs1927906
	rs7037117
	rs5030717
	rs12377632
	rs4986791
TLR6	rs7673124
	rs1039559
	rs2174284
VEGFA	rs833069
	rs3025033
	rs699947
	rs2146323

WNT5A	rs1829556
	rs11706227
	rs524153

Supplementary Table S2: Distribution of selected markers in the pooled group of stage II-III colorectal patients treated with adjuvant fluoropyrimidines-based therapy (n=270).

**Gene**	**Polymorphism**	**Genotype**	**Pooled population**	
			**n**	**(%)**
SMAD3	rs11636161	GG	114	42.2%
		GA	109	40.4%
		AA	47	17.4%
SMAD3	rs1545161	AA	114	42.4%
		AG	116	43.1%
		GG	39	14.5%
FOXO3	rs12203787	GG	204	75.8%
		GC	60	22.3%
		CC	5	1.9%
IFNG	rs1861494	AA	183	68.0%
		AG	74	27.5%
		GG	12	4.5%
VEGFA	rs2146323	CC	116	44.4%
		CA	120	46.0%
		AA	25	9.6%
TGFBR1	rs928180	AA	232	86.2%
		AG	36	13.4%
		GG	1	0.4%
TGFBR2	rs1346907	AA	80	30.1%
		AG	131	49.2%
		GG	55	20.7%
STAT5A	rs7217728	AA	125	46.8%
		AG	118	44.2%
		GG	24	9.0%
STAT5B	rs8080122	GG	123	46.8%
		AG	118	44.9%
		AA	22	8.4%

Supplementary Figure S1: Multiparametric score of disease free survival (DFS) in the stage III patients according to an increasing number of clinical (gender, tumor site) and genetic (*MTHFR*-rs1801131, *IFNG*-rs1861494) risk factors.

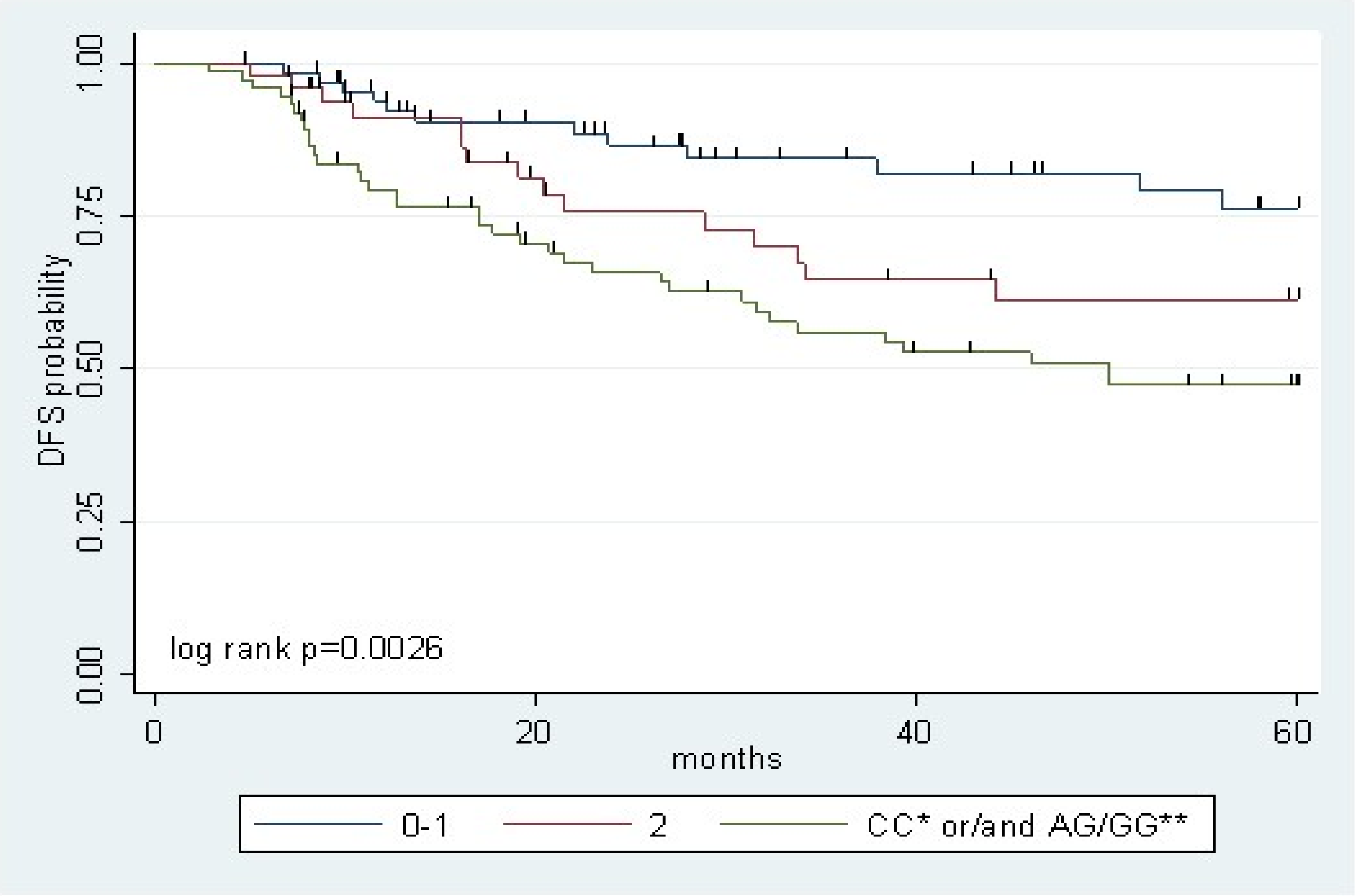

* refers to *MTHFR*-rs1801131 variant
** refers to *IFNG*-rs1861494 variant
